# Naïve human iPSCs obtained by culturing the ICGi022-A cell line with primed pluripotency in HENSM medium efficiently differentiate into endothelial derivatives

**DOI:** 10.18699/vjgb-26-19

**Published:** 2026-04

**Authors:** M.A. Arssan, A.I. Shevchenko, S.M. Zakian, I.S. Zakharova

**Affiliations:** Institute of Cytology and Genetics of the Siberian Branch of the Russian Academy of Sciences, Novosibirsk, Russia; Institute of Cytology and Genetics of the Siberian Branch of the Russian Academy of Sciences, Novosibirsk, Russia; Institute of Cytology and Genetics of the Siberian Branch of the Russian Academy of Sciences, Novosibirsk, Russia; Institute of Cytology and Genetics of the Siberian Branch of the Russian Academy of Sciences, Novosibirsk, Russia

**Keywords:** primed and naïve human pluripotent stem cells, directed endothelial differentiation, hereditary disease cellular models, праймированные и наивные плюрипотентные стволовые клетки человека, направленная эндотелиальная дифференцировка, клеточные модели наследственных заболеваний

## Abstract

Naïve human pluripotent stem cells (PSCs) are a promising new tool in biomedical research. They provide access to the early embryonic development programmes and offer breakthrough solutions in regenerative medicine. However, the current inability to obtain long-term cultures of genetically and epigenetically stable naïve human PSC lines poses a challenge to their effective application in biomedicine. The recently proposed HENSM culture medium is claimed to enable the obtaining and long-term maintenance of naïve PSC lines. In this study, the potential of the HENSM medium for obtaining stable naïve human PSC lines was investigated. We successfully reset the primed induced pluripotent stem cell (iPSC) line ICGi022-A (K7-4Lf), derived from a healthy donor, to a naïve state using the HENSM medium. Naïve iPSCs grow in the form of dome-shaped colonies, both with and without a feeder layer of cells. The resulting cells retained expression of the key pluripotency factors and activated the naïve PSC transcriptional programme, including expression of endogenous retroviral elements, early epiblast marker genes and genes associated with totipotency. The naïve iPSC line was capable of differentiating into derivatives of the three primary germ layers, as well as producing trophoblast derivatives. Culturing naïve iPSCs in low-adhesion conditions resulted in the spontaneous formation of three-dimensional structures (blastoids) resembling early human blastocysts. The X chromosome, which was in an eroded inactive state in the original cell line, was reactivated in the naïve cells, but returned to its normal inactive state when the naïve cells were re-primed. Notably, naïve iPSCs demonstrated limited ability to directly differentiate into endothelial cells. However, their competence to give rise to mature endothelial derivatives was restored upon returning to the primed state, achieving comparable efficiency to the original primed iPSCs. Thus, the resulting naïve iPSC line has significant potential for studying the early stages of embryogenesis and for other biomedical applications, including disease modelling. However, the naïve ICGi022-A line proved to be karyotypically unstable during long-term cultivation using HENSM medium. As there is a risk of karyotypic aberrations during the maintenance of naïve PSCs, further improvement of the culture conditions is necessary to obtain reliable, karyotypically stable lines of naïve pluripotent cells.

## Abstract (ru)

Наивные плюрипотентные стволовые клетки (ПСК) человека – новый многообещающий инструмент биомедицинских исследований, открывающий доступ к ранним программам эмбрионального развития и прорывным решениям задач регенеративной медицины. Препятствием на пути к эффективному внедрению наивных ПСК в биомедицину является отсутствие возможности получать длительно культивируемые генетически и эпигенетически стабильные линии наивных ПСК. Ранее было заявлено, что культуральная среда HENSM позволяет индуцировать и длительно поддерживать линии наивных плюрипотентных стволовых клеток человека. В рамках данной работы мы проверили возможность получать стабильные линии наивных ПСК с использованием среды HENSM. С помощью среды HENSM мы успешно перевели линию ICGi022-A (K7-4Lf) праймированных индуцированных плюрипотентных стволовых клеток (ИПСК) здорового донора в наивное состояние. Наивные ИПСК растут в форме сфероподобных колоний как на питающем слое клеток, так и без него. Полученные клетки сохранили экспрессию ключевых факторов плюрипотентности и одновременно активировали транскрипционную программу наивных ПСК, включающую экспрессию эндогенных ретровирусных элементов, генов-маркеров раннего эпибласта и генов, ассоциированных с тотипотентностью. Наивная линия ИПСК была способна дифференцироваться в производные трех первичных зародышевых листков, а также давать производные трофобласта. При культивировании наивных ИПСК в низкоадгезивных условиях наблюдалось спонтанное формирование трехмерных структур – бластоидов, морфологически напоминающих ранние бластоцисты человека. Х-хромосома, имевшая нарушения в неактивном статусе в исходной линии клеток, реактивировалась в наивных клетках и восстанавливала нормальное неактивное состояние при повторном переводе наивных клеток в праймированное состояние. Важно отметить, что наивные ИПСК продемонстрировали низкую способность к прямой направленной дифференцировке в эндотелиоциты, однако их компетентность давать зрелые эндотелиальные производные восстанавливалась после их возврата к праймированному состоянию, достигая эффективности, сопоставимой с таковой у исходных праймированных ИПСК. Таким образом, полученная линия наивных ИПСК обладает значительным потенциалом для исследования ранних стадий эмбриогенеза и других биомедицинских приложений, включая моделирование заболеваний. Тем не менее при длительном культивировании с использованием среды HENSM наивная линия ICGi022-A оказалась кариотипически нестабильной. Поскольку риск кариотипических аберраций при поддержании наивных ПСК сохраняется, в дальнейшем для получения надежных, кариотипически стабильных линий наивных плюрипотентных клеток необходимо совершенствование условий культивирования.

## Introduction

Cultures of human pluripotent stem cells (hPSCs) can reflect various stages of embryonic development. The majority of hPSC lines obtained and used to date are in a primed state, resembling the post-implantation epiblast. These cells are stable in long-term cultivation and are widely used in biomedical research and clinical trials. However, they exhibit limited developmental and differentiation pathways, demonstrating the epigenetic memory phenomenon that biases differentiation towards specific lineages (Stadtfeld, Hochedlinger, 2010; Gafni et al., 2013; Valamehr et al., 2014; Lee et al., 2017; Hu et al., 2020; Yu et al., 2021; Dekel et al., 2022). They cannot be used to model the earlier stages of embryonic development, such as X chromosome inactivation, and their chromatin is more closed, making them less accessible to genome editing by programmable nucleases. They also often demonstrate impaired X chromosome inactivation, termed erosion (Mekhoubad et al., 2012). In contrast, naïve hPSCs are in a more primitive developmental state that closely mirrors the pre-implantation epiblast. This gives them broader developmental plasticity than conventional (primed) hPSCs (Theunissen et al., 2016; Collier, Rugg‐Gunn, 2018; Rostovskaya, 2022). This unique plasticity includes the ability to contribute to both the embryonic and extraembryonic lineages (Theunissen et al., 2014, 2016; Pham et al., 2022), enhanced proliferation and improved clonal expansion through tolerance of single-cell passaging. Greater amenability to genome editing and erased epigenetic memory through reduction of repressive chromatin marks also render naïve hPSCs a promising platform for advancing regenerative medicine, disease modelling and the study of early human development. Recent advances have demonstrated the feasibility of resetting primed hPSCs to the naïve state in defined media composed small molecules and growth factors (Chan et al., 2013; Gafni et al., 2013; Takashima et al., 2014; Theunissen et al., 2014; Ware et al., 2014; Duggal et al., 2015; Zimmerlin et al., 2016; Guo et al., 2017; Lee et al., 2017; Szczerbinska et al., 2019; Bayerl et al., 2021; Khan et al., 2021; Buckberry et al., 2023). Despite these advantages, deriving and maintaining naïve hPSCs in the long term remains challenging. A major concern is the genomic and epigenetic instability reported in naïve cultures, particularly under prolonged or suboptimal conditions. Chromosome aberrations, karyotype abnormalities, and variable X-chromosome reactivation status have all been observed (Theunissen et al., 2014, 2016; Fischer et al., 2025). These vulnerabilities underscore the need for well-defined, optimized culture systems.

The recently developed HENSM medium shows promise in supporting the long-term stable propagation of naïve hPSC lines while preserving the key molecular and functional features of naïve pluripotency (Bayerl et al., 2021). During the medium formulation, it was recognised that WNT signalling activity was the main cause of cell heterogeneity, imprinting loss, and chromosomal rearrangements in naïve hPSC cultures. Thus, the medium lacks WNT activators, such as GSK3 kinase inhibitors, and ensures almost complete downregulation of WNT signalling through tankyrase inhibition. The medium also contains LIF growth factor and MEK kinase inhibitor, two components that are common to all naïve conditions. LIF activates JAK/STAT3 signalling pathway, which is necessary for naïve pluripotency. The MEK inhibitor, together with the PKC and SRC kinase inhibitors in the HENSM medium, downregulates the MEK/ERK cascade, which is responsible for primed pluripotency. The medium was tested on several ESC and iPSC cell lines. It was claimed that HENSM helps preserve genomic imprints and allows long-term naïve hPSC maintenance, which is crucial for accurate disease modelling and developmental studies. However, there are still no reports of long-term cultured naïve hPSCs obtained in the HENSM medium.

The aim of this study is to obtain stable naïve induced pluripotent stem cells (hiPSCs) in HENSM medium by deriving them from an existing primed hiPSC line, ICGi022-A (K7-4Lf ), from a healthy donor (Malakhova et al., 2020). The primed K7-4Lf line is widely used for disease modeling, drug screening, and stem cell research due to its well-characterized genetic background and robust pluripotency (Malakhova et al., 2020; Klepikova et al., 2022; Ustyantseva et al., 2022; Zakharova et al., 2022, 2024; Grigor’eva et al., 2023, 2024; Kopytova et al., 2023; Sheveleva et al., 2023, 2024; Yarkova et al., 2024; Pavlova et al., 2024a, b; Rezvova et al., 2024; Nadtochy et al., 2025). Reprogramming K7-4Lf cells into a naïve state provides an opportunity to evaluate the developmental and differentiation capabilities of naïve pluripotency.

In this study, we reset primed hiPSCs into the naïve state and examined their developmental potential and differentiation abilities. We successfully derived naïve hiPSCs that exhibited key features of early embryonic cells, including spontaneous blastoids formation. Additionally, we investigated the ability of naïve hiPSCs to give rise to differentiated derivatives, with a particular focus on endothelial cells. Furthermore, we analysed the dynamics of X chromosome regulation, focusing on H3K27me3 and XIST RNA as indicators of epigenetic fidelity. Our findings emphasise the functional benefits and epigenetic challenges of naïve pluripotency, highlighting the necessity of improved resetting and culture protocols to maintain genomic and epigenetic stability.

## Materials and methods

hiPSCs cultivation and differentiation. The human induced pluripotent stem cell line ICGi022-A (hPSCreg identifier: RRID:CVCL_ZE02) (Malakhova et al., 2020) with a karyotype of 46, XX was utilised in this study. The ICGi022-A line was derived and maintained under conditions typical of primed pluripotent cells on a layer of mitotically inactivated mouse embryonic fibroblasts. The medium was composed of DMEM/F12 (1:1), with the addition of 15 % serum replacement KnockOut SR (Thermo Fisher Scientific), 1 mM GlutaMax (Thermo Fisher Scientific), 1 % non-essential amino acid solution (NEAA) (Thermo Fisher Scientific), 50 U/ml penicillin and 50 μg/ml streptomycin (InvivoGen), as well as 0.25 mM 2-mercaptoethanol (Thermo Fisher Scientific) and 10 ng/ml bFGF (Sci-store).

Complete HENSM medium (Bayerl et al., 2021), for induction and maintenance of naïve state, consisted of a 1:1 mixture of Neurobasal and DMEM-F12 media, supplemented with 1 % N2, 1 % B27, 1 mM GlutaMAX, 1 % NEAA (all Thermo Fisher Scientific), 50 U/ml penicillin and 50 μg/ml streptomycin (InvivoGen), 0.2 % Geltrex (Thermo Fisher Scientific), 50 μg/ml L-ascorbic acid 2-phosphate (Sigma-Aldrich), 20 ng/ ml recombinant LIF (Sci-store), and kinase inhibitors: 1 μM GSKi PD0325901, 2 μM TNKi XAV939, 2 μM PKCi Gö6983, 1.2 μM SRCi CGP77675 (all R&D) and 5 μM ROCKi thiazovivin (Stemolecule).In order to expand cell cultures, colonies of both naïve and primed pluripotent cells were dissociated using a TrypLE enzyme (Thermo Fisher Scientific) and subsequently transferred into fresh medium containing 10 μM of the ROCKi thiazovivin (Stemolecule). The seeding ratio was established at 1:10 for primed iPSCs and at 1:3 for naïve iPSCs.

The spontaneous differentiation of naïve hiPSCs into derivatives of three primitive germ layers was performed in a monolayer. To achieve this, the cells were seeded at 30 % confluence on Matrigel (Corning), and the HENSM medium was replaced with a differentiation medium containing a 1:1 DMEM/F12 mixture (Gibco), 10 % fetal bovine serum (FBS) (Cell Technologies), 1 mM GlutaMax (Thermo Fisher Scientific), 1 % NEAA solution (Thermo Fisher Scientific), 50 U/ ml penicillin and 50 μg/ml streptomycin (InvivoGen). The medium was changed every other day. The results of the spontaneous differentiation were analysed after 23 days using immunofluorescence.

The directed differentiation protocol for trophoblast derivatives started with the dissociation of confluent naïve IPSC colonies using TrypLE, followed by seeding at a density of 2×106 cells on a 10 cm2 culture surface treated with collagen IV (Sigma-Aldrich) in HENSM medium. After two days, TSC medium was added to the cells as described in (Okae et al., 2018). The TSC medium comprises a 1:1 DMEM/F12 mixture (Gibco), 0.2 % FBS (Cell Technologies), 0.3 % BSA (Sigma-Aldrich), 0.1 mM 2-mercaptoethanol (Sigma-Aldrich), 1 % ITS-X (Gibco), 1.5 μM ascorbic acid (Sigma-Aldrich), 50 ng/ml EGF (Peprotech), 2 μM CHIR99021 and 0.5 μM A83-01, 1 μM SB431542 (all R&D), 0.8 mM valproic acid (Sigma-Aldrich), 5 μM thiazovivin (Stemolecule), 50 U/ml penicillin and 50 μg/ml streptomycin (InvivoGen). The medium was changed daily. On day 5 after the addition of the TSC medium, once the flattened colonies had reached 50–70 % confluence, they were expanded using TrypLE enzymatic disaggregation at a ratio of 1:4. Homogeneous trophoblast cell cultures were formed by the fifth passage. Thereafter, trophoblast cells were passaged with TrypLE every three to four days at a ratio of 1:4 to 1:6. At the tenth passage, the cells were plated on coverslips and stained with antibodies for specific markers.

Endothelial derivatives of hPSCs were obtained through mesodermal progenitor derivation and subsequent endothelial differentiation, according to the Gu protocol with modifications (Gu, 2018). First, the pluripotent cells were seeded onto a matrigel-treated surface (Corning) at a confluence of 60–70 %, in the original iPSC culture medium. The next day, differentiation in the mesodermal direction was started in RPMI 1640 medium containing 1 % B27 supplementation without insulin (all Thermo Fisher Scientific), 50 U/ml penicillin, 50 μg/ml streptomycin (InvivoGen), and the GSK3 kinase inhibitor CHIR99021 (R&D). The concentration of CHIR99021 in the differentiation medium was 6 μM for two days, after which it was reduced to 3 μM for the following two days. The differentiating cells were then transferred to EGM-2 medium (Lonza), supplemented with 50 ng/ml VEGF, 25 ng/ml bFGF (both Sci-Store) and 10 μM SB431542 (R&D), for endothelial growth. For the next eight days, half of the endothelial medium were changed daily. The proportion of cells containing the CD31 marker of mature endothelial cells was determined using a FACSAria III device (BD, USA) after precipitating the cells with the appropriate antibodies (Supplementary Material 1)1 and analysing 104 events. Unstained cells and cells incubated with fluorescently labelled mouse IgG1 were used as negative controls. The experiment was repeated three times. The statistical significance of the differences was measured using the Wilcoxon test with Bonferroni correction. Mature endothelial derivatives of hiPSCs were selected using magnetic sorting with antibodies to the human CD31 surface antigen (Miltenyi Biotec) and seeded onto a collagen IV-coated culture surface (Sigma-Aldrich) in EGM2 medium (Lonza).

Supplementary Materials are available in the online version of the paper: https://vavilovj-icg.ru/download/pict-2026-30/appx12.pdf


Generation of human blastoids from naïve human iPSCs maintained in HENSM medium. For the generation of blastoids, naïve hiPSCs with 60–70 % confluence were dissociated into single cells using TrypLE Express at 37 °C for 3 minutes. The cells were then collected by centrifugation at 200g for 5 minutes and resuspended in fresh HENSM medium. The resulting cell suspension was incubated in a gelatinised tissue culture plate for 30 minutes at 37 °C in 5 % CO2 to remove the feeder cells. The medium containing naïve human iPSCs was collected and passed through a 40 μm cell strainer. The AggreWell 400 (STEMCELL Technologies) was prepared by rinsing with an anti-adhesion solution (STEMCELL Technologies), followed by centrifugation at 2,000g for 5 minutes and incubation at room temperature for 10 minutes, according to the manufacturer›s instructions. After incubation, wells were washed once with HENSM medium, after which 0.5 ml of fresh HENSM containing 5 μM hiazovivin (Stemolecule) was added.

Approximately 30,000 cells (around 25 cells per microwell) were resuspended in 1 ml HENSM with 5 μM thiazovivin (Stemolecule) and seeded into one well of a prepared AggreWell 400 24-well plate. The plate was centrifuged at 200g for 1 minute and placed at 37 °C. Aggregates were formed within 12–16 hours. The HENSM medium was then changed to TDM medium and replaced every two days for six days. The TDM previously described for 5iLA-naïve hPSCs was slightly modified to adjust the HENSM protocols using the following: 1:1 (v/v) mixture of DMEM/F12 and Neurobasal medium, 0.5× N2 supplement, 0.5 % 0.5× GlutaMAX, 0.5× non-essential amino acids (all Thermo Fisher Scientific), 0.5× B27 supplement (Thermo Fisher Scientific), 0.1 mM β-mercaptoethanol (Sigma-Aldrich), 1 % ITS-X (Gibco), 0.5 % KnockOut SR (Thermo Fisher Scientific), 0.1 % FBS (Cell Technologies), 50 mg/ml BSA (Sigma-Aldrich), 50 U/ ml penicillin and 50 μg/ml streptomycin (InvivoGen), 1 μM Gö6983, 1 μM PD0325901, 0.5 μM CGP77675, 0.5 μM A83-01, 1.25 μM CHIR99021, 0.5 μM SB431542 (all R&D), 25 ng/ml recombinant human LIF (Sci-Store), 10 ng/ml EGF (Peprotech), 0.75 μg/ml L-ascorbic acid, and 0.4 mM valproic acid (both from Sigma-Aldrich). After six days, the blastoids were isolated and treated with a 4 % formaldehyde fixative solution in PBS for immunostaining with OCT4 antibody.

RNA isolation, cDNA synthesis and semi-quantitative real- time PCR. RNA was isolated from primed and naïve iPSCs using a TRIzol reagent (Thermo Fisher Scientific) and a DNA-free kit (Thermo Fisher Scientific). cDNA was synthesised using a reverse transcriptase (M-MuLV) (Biolabmix) and a random hexamer primer (Thermo Fisher Scientific), according to the manufacturers’ instructions. The relative gene expression levels of NANOG, TFCP2L1, KLF17, TFE3, LTR7Y, DAZL and LEUTX were determined by semi-quantitative real-time PCR using the ΔΔCt method, normalised to the levels of the housekeeping genes ACTB, TFRC and B2M. The experiment was performed in three biological and two technical replicates. The results were analysed using the qBase+ software (CellCarta, https://cellcarta.com/genomic-data-analysis/) and the generalised ΔΔCt method was employed, taking into account the reaction efficiency, which was calculated from the calibration curve results. The sequences of the oligonucleotides synthesised at Biosset (https://www.biosset.com/) are given in the Supplementary Material 2.

Immunofluorescent staining and RNA FISH. We used indirect immunofluorescence staining to detect surface antigens, transcription factors, and the epigenetic state of the X chromosome in hiPSCs, as well as markers of their differentiated derivatives. During cell preparation and antibody staining, we follow the protocol described previously (Vaskova et al., 2015; Zakharova et al., 2017, 2022). The cells were fixed in a 4 % formaldehyde solution for 10 minutes, permeabilised with a 0.5 % Triton X-100 solution (Sigma-Aldrich) for 30 minutes (this step was omitted for surface antigens), and blocked with a 1 % bovine serum albumin solution in 1x PBS. All procedures were performed at room temperature. Primary antibody incubation was conducted overnight at 4 °C. Precipitation between cells and fluorescently labelled secondary antibodies was performed in the dark at room temperature for one hour. Cell nuclei were stained with DAPI. The preparations were visualized and imaged using a Ti-E inverted fluorescence microscope (Nikon) and NIS Advanced Research software. Antibodies, their manufacturers, species of origin, and dilutions are listed in Supplementary Material 2

To examine the epigenetic state of the X chromosome, immunofluorescent staining with antibodies to chromatin modification of the inactive X chromosome was combined with RNA FISH with probes to the RNA XIST (BAC clone RP11-256P2) and X-linked gene HUWE1 (BAC clone RP11- 975N19), according to the protocol given in (Vaskova et al., 2015). Images were acquired and analysed using a Nikon Ti-E inverted fluorescent microscope (Japan) and NIS Elements AR software. At least 300 nuclei were analysed in each experiment.

STR analysis. The short tandem repeat (STR) profile of the primed and naïve PSC line was determined by polymerase chain reaction with the COrDIS EXPERT 26 reagent kit (Genoanalytica, https://www.genoanalytica.ru/) according to the manufacturer’s protocol, followed by amplicon separation on a 3130 Genetic Analyzer capillary electrophoresis instrument (Applied Biosystems). Electropherograms of the STR profiles obtained by PCR are available upon request from the authors. Table with STR results is provided in Supplementary Material 3.

Mycoplasma and episome detection. The absence of mycoplasma and episome contamination in the cells was detected by PCR. The primer sequences are provided in the Supplementary Material 2. Parameters for episome detection: 95 °C for 5 minutes, followed by 35 cycles of 95 °C for 15 seconds, 58 °C for 15 seconds and 72 °C for 20 seconds, then 72 °C for 5 minutes. Parameters for mycoplasma detection: 95 °C for 3 minutes, followed by 35 cycles of 95 °C for 15 seconds, 67 °C for 15 seconds and 72 °C for 20 seconds, then 72 °C for 5 minutes.

Karyotyping. Naïve hiPSCs were karyotyped at passages 12, 15 and 18. Metaphase chromosome spreads were obtained by methanol-acetic acid fixation, as previously described (Grigor’eva et al., 2024). The karyotype of naïve iPSCs was determined according to the International System of Human Cytogenetic Nomenclature

## Results

**Fig. 1.**
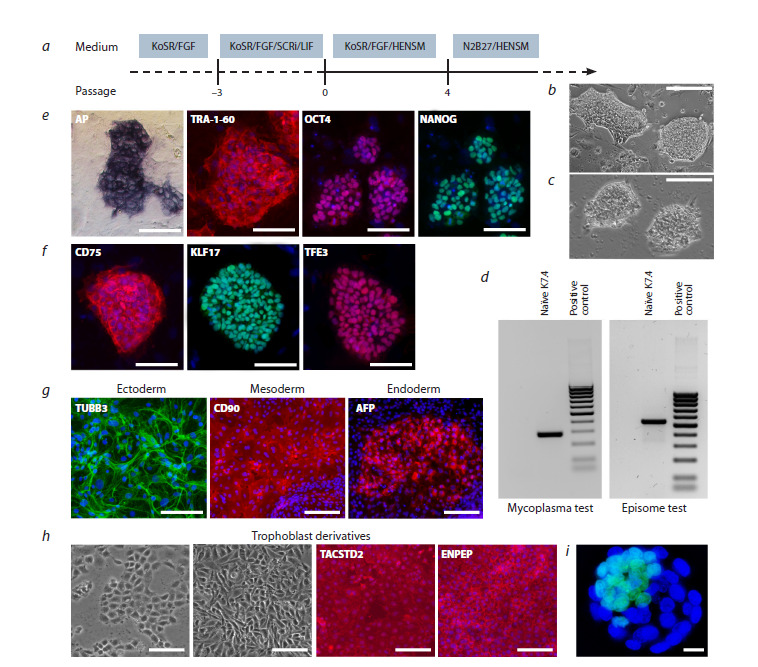
K7-4Lf hiPSCs after induction of naïve pluripotency in HENSM medium. a – schematic representation of the conversion stages from primed to naïve state; b – colony morphologies of naïve hiPSCs on a layer of mitotically inactivated mouse embryonic fibroblasts, Scale bar is 100 μm; c – colony morphologies of naïve hiPSCs on Matrigel-treated surface; Scale bar is 100 μm; d – absence of episomal vectors and mycoplasma contamination in naïve K7-4Lf line; e – common pluripotency markers: alkaline phosphotase (AP), TRA-1-60 (red), OCT4 (red), NANOG (green) detected in naïve hiPSCs line. Nuclei are stained with DAPI (blue). Scale bar is 100 μm; f – specific markers of naïve pluripotency: surface antigen CD75 (red), transcription factors KLF17 (green) and TFE3 (red). Nuclei are stained with DAPI (blue). Scale bar is 100 μm; g – ability of naïve iPSC line to differentiate into derivatives of three germ layers; h – morphology of early trophectoderm cells derived from naïve hiPSCs and immunostaining with antibodies to early placentation markers TACSTD2 (red) and ENPEP (red). Nuclei are stained with DAPI (blue). Scale bar is 100 μm; i – representative image of blastoid obtained by self-aggregation of naïve K7-4Lf iPSCs under low-adhesive conditions. Рluripotent cells are stained green with antibodies to OСT4. Nuclei are stained with DAPI. Scale bar is 10 μm.

Several media have been proposed to date that allow primed human PSCs to be reset to a naïve state (Chan et al., 2013; Gafni et al., 2013; Takashima et al., 2014; Theunissen et al., 2014; Ware et al., 2014; Duggal et al., 2015; Zimmerlin et al., 2016; Guo et al., 2017; Lee et al., 2017; Szczerbinska et al., 2019; Bayerl et al., 2021; Khan et al., 2021; Buckberry et al., 2023). Among these, the HENSM medium has been shown to capture a naïve state that closely resembles the early preimplantation epiblast (Bayerl et al., 2021). It has been claimed that naïve PSC lines obtained with the HENSM protocol are karyotypically and epigenetically stable during long-term cultivation. In this study, we aimed to return a primed hiPSC line to a naïve state using HENSM conditions. We used K7-4Lf iPSCs that were obtained and described previously (Malakhova et al., 2020). However, direct transferring K7-4Lf cells from a DMEM/F12 medium supplemented with KnockOut SR and FGF2 into a complete HENSM medium resulted in extensive cell death, preventing further progression of the resetting process. To overcome this barrier, we adopted a stepwise approach (Fig. 1a), first culturing the K7-4Lf cells in a primed cell medium supplemented with an SRC kinase inhibitor (CGP77675) and LIF. This pre-conditioning phase was lasted for three passages before the cells were transferred into a DMEM/F12-based HENSM naïve-induction medium that still omitted N2B27 supplements but contained KnockOut SR and FGF2, as well as the complete HENSM small molecule set and growth factor LIF. By the fourth passage in the naïve-inducing medium, the K7-4Lf colonies had begun to exhibit a transition towards the compact, dome-shaped morphology characteristic of naïve-like human pluripotent stem cells (Fig. 1b). At this stage, the cultures were transferred to complete N2B27-based HENSM medium supplemented with Geltrex. Under these conditions, the cells exhibited stable growth and could be propagated enzymatically using TrypLE in the presence of a ROCK inhibitor.

Following the establishment of the naïve culture, we removed FGF2 from the N2B27-based HENSM medium. While FGF2 supports initial cell survival during resetting, its continued presence can hinder the efficient acquisition of the naïve state. Notably, the resulting K7-4Lf cells maintained hallmark features of naïve pluripotency after FGF2 withdrawal at passage 6 of the resetting process, indicating that they had achieved FGF2-independent pluripotency. Furthermore, these cells were capable of forming compact, spherical colonies under feeder-free conditions when cultured on Matrigel-coated plates (Fig. 1c). However, during the early stages of resetting to the naïve state, we observed that K7-4Lf cells failed to maintain under feeder-free conditions, even in complete HENSM medium. This suggests a transient dependency on FGF2 and/or feeder support during the initial phases of resetting to the naïve state.

The resulting naïve K7-4Lf cells demonstrated robust stability under enzymatic passaging and could be cryopreserved and thawed without any apparent loss of viability or naïve morphology. The naïve K7-4Lf line maintained as mycoplasma free culture (Fig. 1d ). It showed no signs of the episomes that had been used to derive the original K7-4Lf, indicating that resetting to a naïve state was free from the expression of exogenous reprogramming factors. We also demonstrated that the original cell line and its naïve derivative have identical STR profiles (see Supplementary Material 3).

**Naïve K7-4Lf hiPSCs characterisation**

The resulting naïve K7-4Lf cells demonstrated characteristics commonly associated with pluripotent cells. A histochemical assay revealed alkaline phosphatase activity (Fig. 1e), and immunostaining confirmed the presence of the transcription factors OCT4 and NANOG, as well as the conventional surface marker TRA-1-60 (Fig. 1e). Additionally, the cells expressed the naïve-specific markers CD75, KLF17, and TFE3 (Fig. 1f ). Notably, TFE3 nuclear translocation is considered a pivotal event in the induction of naïve pluripotency and a defining feature of the naïve pluripotent state (Gafni et al., 2013; Mathieu et al., 2019). Functionally, these cells were able to differentiate into derivatives of all three germ layers (Fig. 1g), as well as into trophoblast cells that expressed surface markers typical of early placentation (Fig. 1h). Furthermore, naïve K7-4Lf cultures exhibited spontaneous self-organisation into blastoid-like structures under low-adhesion conditions (Fig. 1i), indicating that the cells possess developmental potency attributable to the naïve pluripotent state.

**Fig. 2.**
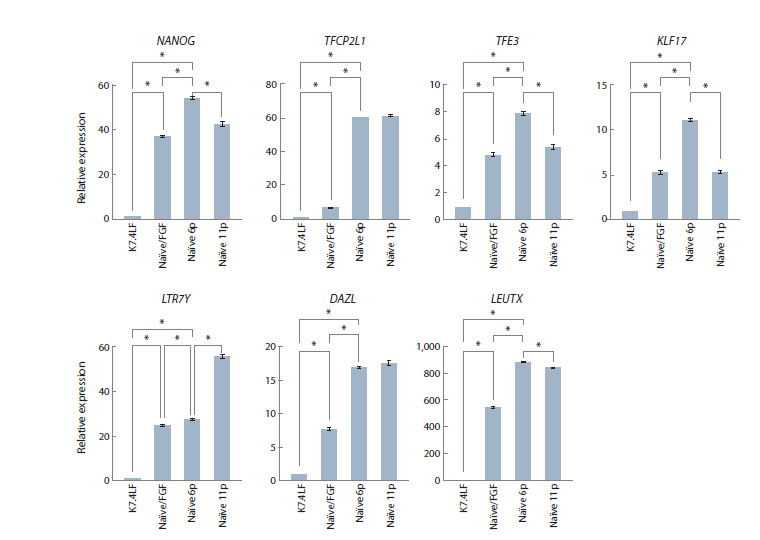
Semi-quantitative analysis of NANOG, TFCP2L1, TFE3, KLF17, LTR7Y, DAZL and LEUTX gene expression in the original primed K7-4Lf line and its naïve derivatives cultured in KoSR/FGF/HENSM (Naïve/FGF) as well as in N2B27/HENSM (Naïve) at 6 and 11 passages. Statistically significant differences (p <0.05) are marked with an asterisk.

Furthermore, we analysed the expression of pluripotencyassociated genes using RT-PCR. We focused specifically on how gene expression differs in naïve K7-4Lf cultures with and without FGF2. To monitor transcriptional dynamics over time, we analysed gene expression profiles at passages 6 and 11. Generally, we found that the naïve K7-4Lf line exhibited robust upregulation of naïve-specific transcription factors compared to the original primed cells (Fig. 2).

Previous studies have demonstrated that NANOG and TFCP2L1 play a significant role in the acquisition and maintenance of the naïve pluripotent state (Theunissen et al., 2016; An et al., 2020). We found that NANOG expression increased substantially in FGF2-independent naïve K7-4Lf cells compared to those maintained with FGF2. Naïve K7-4Lf cultures also demonstrated concomitant TFCP2L1 upregulation. In line with previous findings that FGF2 impairs the efficient resetting to naïve pluripotency, TFCP2L1 levels were significantly higher in the FGF2-free condition. NANOG expression increased through passage 6, but then declined by passage 11. In contrast, TFCP2L1 expression remained consistently high across both time points. The expression patterns of KLF17 and TFE3 mirrored the dynamic profile of NANOG, indicating coordinated regulation during the maturation of naïve identity.

In vivo studies of the early epiblast showed marked activity of transposable elements, particularly HERVH and LTR7Y (Szczerbinska et al., 2019). Mounting evidence implicates these endogenous retroviral elements as functional regulators of early human development. Consistent with this, we observed robust LTR7Y expression in naïve K7-4Lf cultures, with transcription levels progressively increasing over passages. This contrasted with the NANOG expression pattern, potentially indicating a transition into a developmental state beyond the NANOG-regulated phase of naïve pluripotency.

The expression of a DAZL gene, which is also known as a naïve hPSCs marker, was also examined. DAZL orchestrates the initial stages of PGC commitment and is involved in the upregulation of TET1, which balances the level of 5-hydroxymethylcytosine (Welling et al., 2015). DAZL expression was elevated in naïve K7-4Lf hiPSCs and remained consistently higher in FGF2-independent naïve cultures. This finding suggests that the cells have acquired a molecular profile conducive to both germline differentiation competence and stable naïve pluripotency. 

Strikingly, the naïve K7-4Lf cells also activated genes associated with a totipotent-like state. Specifically, we observed increased expression of the LEUTX gene, which encodes a chromatin protein involved in zygotic genome activation at the eight-cell stage (Mazid et al., 2022), with higher levels detected in FGF2-independent cultures. This is in line with our previous findings and suggests that the removal of FGF2 enables a more advanced reprogramming trajectory towards earlier embryonic-like states (Shevchenko et al., 2025).

**Fig. 3.**
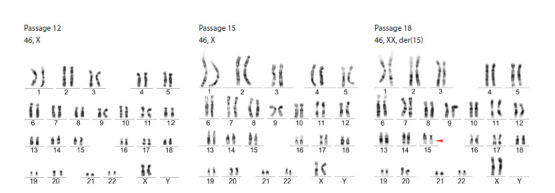
Naïve hiPSCs K7-4Lf karyotyping at passages 12, 15 and 18.

In parallel with transcriptional analyses, we monitored chromosomal stability across passages. Karyotype analysis of naïve K7-4Lf cells at passage 12 revealed a normal chromosomal complement (Fig. 3). However, by passage 15, we detected signs of aneuploidy and polyploidy, indicating chromosomal instability during long-term cultivation. The subsequent karyotype analysis at passage 18 revealed a deletion in the short arm of chromosome 15 (Fig. 3) in more than 70 % of cells. This highlights the importance of ongoing genomic surveillance during long-term culture and suggests that medium composition and culture conditions can affect the karyotype stability in naïve hPSCs obtained and maintained in HENSM.

**X chromosome epigenetics in primed, naïve and re-primed K7-4 iPSCs**

In primed hPSC lines with two X chromosomes, one is active (Xa) and the other is inactive (Xi) due to X chromosome inactivation (XCI), which is complete by this developmental stage (Vallot et al., 2015; Disteche, 2016; Patel et al., 2017; Sahakyan et al., 2017). However, in most primed hPSC lines, the inactive state on Xi often becomes disrupted and undergoes erosion (Xe). XCI erosion is characterised by the loss of inactive chromatin marks and the partial reactivation of genes, preventing Xe from being re-inactivated during differentiation. This erosion is undesirable for hPSC application in biomedical research. Some naïve media, including HENSM, enable pluripotent cells to restore an inactive state on Xe when the cells are returned to a primed state and differentiated into germinal and somatic cells (Sahakyan et al., 2017; Vallot et al., 2017; An et al., 2020; Raposo et al., 2025). In this study, we evaluated the epigenetic state of the X chromosome in the original primed hiPSC line K7-4Lf, focusing on the changes that occur when the cells are reset to the naïve state and when the naïve cells are returned to the primed state (re-primed).

**Fig. 4.**
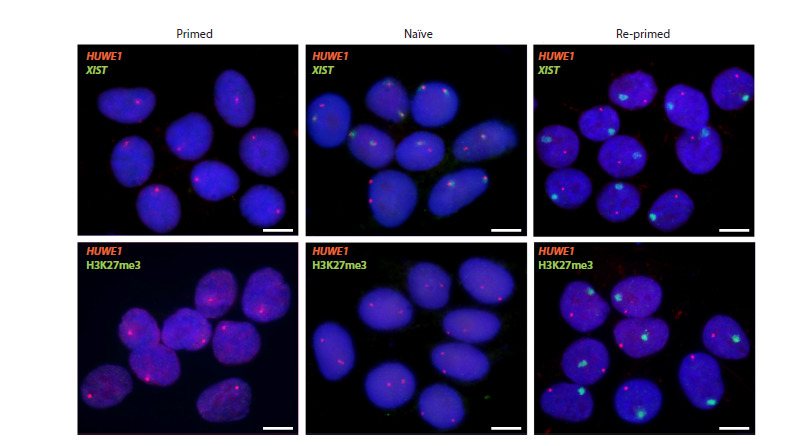
X chromosome epigenetic state in the original primed, naïve and re-primed K7-4Lf hiPSCs, detected bу RNA FISH with probes to RNA XIST (green) and HUWE1 gene (red) as well as immunostaining to H3K27me3 histone mark (green). Nuclei are counterstained with DAPI (blue). Scale bar is 10 μm.

Using RNA FISH and immunofluorescence, we showed that in the original primed K7-4Lf hiPSC line, 96.0 ± 1.2 % of nuclei lacked XIST RNA and H3K27me3 histone modification clouds, which are attributable to the Xi territory and are main XCI participants (Fig. 4). Thus, it can be stated that erosion on the inactive X chromosome is observed in the original K7- 4Lf line. However, in primed K7-4Lf hiPSCs, the majority of cells (96.5 ± 2.1 %) exhibited just one HUWE1 signal per nucleus, suggesting that it is transcribed on Xa and remains inactive on Xe. Reactivation of HUWE1 on Xe is considered a late erosion event, suggesting that XCI erosion in the original K7-4Lf line is at an early stage (Vallot et al., 2015; Patel et al., 2017; Raposo et al., 2025)Naïve K7-4Lf cells cultured in HENSM medium show biallelic expression of the X-linked gene HUWE1 in 95.0 ± 1.6 % of nuclei. Biallelic expression of XIST RNA is also detected in some cells (27.5 ± 2.1 %). Accumulation of the inactive H3K27me3 modification on X chromosomes was not detected. This epigenetic state is typical of naïve hPSCs and reflects the intermediate stages of the X-chromosome reactivation that is characteristic of the early pluripotent state in humans (Vallot et al., 2015; Theunissen et al., 2016; Sahakyan et al., 2017; Bayerl et al., 2021; Khan et al., 2021).

In K7-4Lf cells derived from naïve hiPSCs after re-priming, we observed pattern of completed XCI (95.5 ± 1.3 % of nuclei). It appears as one distinct RNA XIST or H3K27me3 cloud corresponding to Xi and as one signal of X-linked gene HUWE1 located apart from, corresponding to Xa (Fig. 4).

Thus, obtaining cells in naïve state from primed K7-4Lf hiPSC line in HENSM medium, and re-priming or differentiating them, allows us to get rid of erosion and obtain a culture with normal XCI.

**Directed differentiation of naïve hiPSCs into endothelial derivatives**

Although naïve pluripotent cells have an expanded differentiation potential, they may not differentiate efficiently into somatic lineages directly (Lee et al., 2017; Rostovskaya et al., 2019; Guo et al., 2021; Buckberry et al., 2023). To achieve efficient differentiation into specific somatic cell types, naïve hPSCs first need to be returned back to the primed state, after which their somatic derivatives can successfully be obtained according to protocols previously established for primed hPSCs.

In this study, we evaluated the effect of naïve pluripotency on the ability of hiPSCs to differentiate into endothelial-like cells (ECs), which had not been investigated previously. We compared the ability of the original primed K7-4Lf line, naïve and re-primed K7-4Lf cells to produce mature endothelial derivatives. Re-priming entails placing naïve K7-4Lf cultures in the medium used for maintaining primed K7-4Lf hiPSCs for 48 hours prior to directed EC differentiation.

**Fig. 5.**
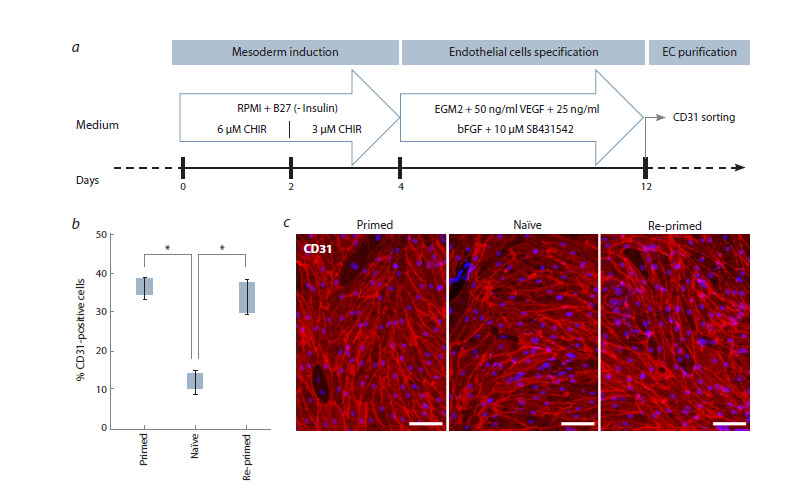
Directed differentiation of original primed, naïve and re-primed K7-4Lf hiPSCs into endothelial derivatives. a – schematic representation of the differentiation protocol; b – quantification of mature EC using CD31 (PECAM1) surface marker. Statistically significant differences (p <0.05) are marked with an asterisk; c – hiPSC derived endothelial cells are stained with antibody to CD31 (red). Nuclei are counterstained with DAPI (blue). Scale bar is 100 μm.

We differentiated primed, naïve and re-primed K7-4Lf cells into ECs using a two-step directed protocol (James et al., 2010; Gu, 2018; Zakharova et al., 2024) (Fig. 5a). It comprises (1) mesoderm induction by WNT pathway activation and subsequent (2) endothelial specification using VEGF and FGF in EGM2 medium along with TGFβ pathway inhibition to suppress smooth muscle cell lineage. On day eight of endothelial specification, we used flow cytometry to quantify mature ECs for the endothelial marker CD31 (PECAM1) (Fig. 5b).

We found that attempts to directly differentiate K7-4Lf cells maintained in a naïve state resulted in poor induction of endothelial derivatives. This suggests that naïve hiPSCs have an impaired or incompatible background for endothelial specification. However, re-primed and primed K7-4Lf cells exhibited a significant proportion of mature endothelial cells, which did not differ significantly from each other and were comparable with endothelial specification experiments performed in other studies (Shevchenko et al., 2023; Zakharova et al., 2024). Thus, we found that re-priming renders the naïve K7-4Lf line competent to differentiate into endothelial cells at a level comparable to the original primed K7-4Lf cells (Fig. 5b).

In conclusion, endothelial cells obtained from all three K7- 4Lf sources through this differentiation protocol by magnetic sorting were positive for CD31 (Fig. 5c), which is consistent with their identity as vascular endothelial cells

## Discussion

The rigorous selection of HENSM medium composition, taking into account the influence of its components on chromosomal, epigenetic and genetic stability, allowed optimal conditions for long-term cultivation of naïve hPSCs to be identified. This potentially paves the way for the mass production of stable naïve hPSC lines and their broader use in biomedical research.

In this study, we successfully reset the primed K7-4Lf hiPSC line to the naïve state using the HENSM medium. The characteristics of the resulting naïve iPSCs were summarised in the preliminary cell line passport (Supplementary Material 4). However, transferring primed hiPSCs directly into HENSM medium resulted in extensive cell loss, hindering efficient reprogramming and the establishment of stable naïve cultures. To address this issue, we optimised the induction protocol by introducing a transitional phase, culturing the hPSCs in SRC kinase inhibitor and LIF-supplemented medium prior to naïve induction. This intermediate step was crucial, as it conferred the necessary competence for naïve induction, reducing to minimum cell death and improving the efficiency and stability of the resetting process. The effectiveness of the transitional stage likely stems from its ability to reset the cellular signalling and epigenetic landscape, thereby priming the cells for subsequent naïve resetting. By modulating key pathways and reducing lineage priming, the transitional culture creates a more permissive environment for acquiring naïve pluripotency. Our optimised protocol, which included enzymatic passaging with TrypLE, consistently generated stable naïve hPSCs. These cells exhibited robust expression of pluripotency markers and significant upregulation of naïve-specific genes. Notably, the naïve hPSCs produced using our protocol exhibited the unique ability to form trophoblast cells and blastoid-like structures in vitro, highlighting their developmental potential and fidelity to the naïve state.

Our naïve K7-4Lf cells showed upregulation of LEUTX, which is a key marker of totipotent-like subpopulations that are active during early embryonic development. The increase in LEUTX expression following the withdrawal of FGF2 is consistent with our previous study (Shevchenko et al., 2025), which found that removing growth factors such as FGF2 increases the number of LEUTX-positive cells. This indicates a shift towards a totipotent-like transcriptional state within naïve hPSC cultures.

Naïve hPSCs demonstrate superior competence in generating neural stem cells, progenitors and primordial germ cell-like cells, compared to their primed counterparts (Irie, Surani, 2016; Kisa et al., 2017; Lee et al., 2017; Ozaki et al., 2022; Buckberry et al., 2023). This enhanced differentiation potential positions naïve hPSCs as a promising platform for various lineage specifications. However, naïve hPSCs appear to be limited in their ability to generate endothelial cells directly in vitro. This phenomenon may be explained by the absence of key transcription factors, such as OTX2 and ZIC2/3, which are essential for initiating gastrulation and subsequent somatic lineage specification. These genes are typically expressed in the post-implantation epiblast, a developmental stage that is mimicked by primed hPSCs (Warr et al., 2008; Di Giovannantonio et al., 2021; Ee et al., 2024; Hossain et al., 2024), but are absent in the naïve state. Consistent with this, we found that re-priming naïve hPSCs restores their ability to differentiate into endothelial cells. Therefore, the ability of naïve hPSCs to generate mesodermal and endodermal derivatives appears restricted unless the cells undergo a transient re-priming process (Guo et al., 2017; Lee et al., 2017; Liu et al., 2017; Buckberry et al., 2023).

The loss of nuclear XIST RNA and H3K27me3 foci in the original K7-4Lf line indicates erosion of the inactive X chromosome. Despite the fact that it occurs frequently, the erosion is often disregarded in stem cell research. XCI erosion leads to the aberrant expression of X-linked genes, which impairs the differentiation potential of hPSCs. This results in abnormal descendants that fail to accurately model physiological conditions (Anguera et al., 2012; Mekhoubad et al., 2012; D’Antonio-Chronowska et al., 2019; Motosugi et al., 2022). Importantly, XCI erosion persists after differentiation, compromising the reliability of disease models and drug screening platforms (Mekhoubad et al., 2012; Vallot et al., 2015; Patel et al., 2017). Differentiated derivatives with erosion may be non-functional and at risk of oncogenic transformation. Therefore, monitoring and preventing XCI erosion is essential for ensuring the fidelity of hPSC-derived models and clinical applications. Resetting to the naïve state enables reactivation of the X chromosome, but this is neither complete nor homogeneous, either between cell lines or between cells within a culture. Contrary to previous findings, a recent study has demonstrated that human pre-implantation epiblasts display two active X chromosomes marked by H3K27me3 (Alfeghaly et al., 2024). These observations suggest that generated naïve hPSCs exist in a metastable, transitional state that does not fully recapitulate the pre-implantation epiblast in vivo (Alfeghaly et al., 2024). Nevertheless, re-primed hiPSCs regained XIST RNA and H3K27me3 foci, indicating re-establishment of the normal inactive X chromosome state.

Chromosomal instability is a well-recognised challenge in naïve hPSC cultures. Previous reports indicate that naïve hPSCs typically retain a normal karyotype for up to nine passages when maintained in 5iLAF medium before abnormalities arise (Theunissen et al., 2014). In our study, naïve hiPSCs generated using our optimised protocol retained normal karyotypes for up to 15 passages, exceeding earlier benchmarks. However, chromosomal abnormalities were detected by passage 18. These results imply that, although our approach increases the period of genomic stability, the risk of karyotypic aberrations continues with prolonged culture. Ongoing monitoring and further refinement of culture conditions are necessary to ensure the genomic integrity of naïve hPSCs for research and therapeutic applications.

## Conclusion

In summary, we successfully reset the primed K7-4Lf hiPSC line to a naïve pluripotent state, using these cells to explore the developmental and functional properties of human naïve pluripotency. Our findings demonstrate the potential of naïve reprogramming in accessing early developmental stages and modelling key aspects of embryogenesis by blastoid formation.We identified the expression of totipotent-like genes within the naïve hPSC cultures, which reinforces the developmental plasticity of this state. In parallel, we demonstrated that re-priming can effectively recapitulate somatic differentiation competence. This enables the efficient generation of endothelial-like cells, thereby underscoring the developmental relevance of transitioning between pluripotent states. Naïve pluripotency opens up new opportunities to control and understand X chromosome reactivation and inactivation epigenetics in humans. However, this topic remains challenging and requires further study. Moving forward, the field must strive to develop more refined, stable and clinically translatable naïve hPSC systems that preserve the benefits of this state while mitigating its disadvantages. As progress continues, naïve hPSCs will remain pivotal in exploring human development and pioneering nextgeneration regenerative therapies

## Conflict of interest

The authors declare no conflict of interest.

## References

Alfeghaly C., Castel G., Cazottes E., Moscatelli M., Moinard E., Casanova
M., Boni J., … Boers J., Gribnau J., David L., Ouimette J.F.,
Rougeulle C. XIST dampens X chromosome activity in a SPENdependent
manner during early human development. Nat Struct Mol
Biol. 2024;31(10):1589-1600. doi 10.1038/s41594-024-01325-3

An C., Feng G., Zhang J., Cao S., Wang Y., Wang N., Lu F., Zhou Q.,
Wang H. Overcoming autocrine FGF signaling-induced heterogeneity
in naïve human ESCs enables modeling of random X chromosome
inactivation. Cell Stem Cell. 2020;27(3):482-497. doi
10.1016/j.stem.2020.06.002

Anguera M., Sadreyev R., Zhang Z., Szanto A., Payer B., Sheridan S.,
Kwok S., … Alvarez J., Gimelbrant A., Mitalipova M., Kirby J.E.,
Lee J.T. Molecular signatures of human induced pluripotent stem
cells highlight sex differences and cancer genes. Cell Stem Cell.
2012;11(1):75-90. doi 10.1016/j.stem.2012.03.008

Bayerl J., Ayyash M., Shani T., Manor Y., Gafni O., Massarwa R.,
Kalma Y., … Hanna S., Ben-Yosef D., Novershtern N., Viukov S.,
Hanna J.H. Principles of signaling pathway modulation for enhancing
human naïve pluripotency induction. Cell Stem Cell. 2021;28(9):
1549-1565.e12. doi 10.1016/j.stem.2021.04.001

Buckberry S., Liu X., Poppe D., Tan J.P., Sun G., Chen J., Nguyen
T.V., …
Breen J., Faulkner G.J., Nefzger C.M., Polo J.M., Lister R. Transient
naïve reprogramming corrects hiPS cells functionally and epigenetically.
Nature. 2023;620(7975):863-872. doi 10.1038/s41586-023-
06424-7

Chan Y.S., Göke J., Ng J.H., Lu X., Gonzales K.A., Tan C.P., Tng W.Q.,
Hong Z.Z., Lim Y.S., Ng H.H. Induction of a human pluripotent state
with distinct regulatory circuitry that resembles preimplantation epiblast.
Cell Stem Cell. 2013;13(6):663-675. doi 10.1016/ j.stem.2013.
11.015

Collier A., Rugg‐Gunn P. Identifying human naïve pluripotent stem
cells − evaluating state‐specific reporter lines and cell‐surface markers.
BioEssays. 2018;40(5):1700239. doi 10.1002/bies.201700239

D’Antonio-Chronowska A., Donovan M.K.R., Young Greenwald W.W.,
Nguyen J.P., Fujita K., Hashem S., Matsui H., … Coulet F.,
Smith E.N., Adler E., D’Antonio M., Frazer K.A. Association of
human iPSC gene signatures and X chromosome dosage with two
distinct cardiac differentiation trajectories. Stem Cell Rep. 2019;
13(5):924-938. doi 10.1016/j.stemcr.2019.09.011

Dekel C., Morey R., Hanna J., Laurent L., Ben-Yosef D., Amir H. Stabilization
of hESCs in two distinct substates along the continuum
of pluripotency. iScience. 2022;25(12):105469. doi 10.1016/j.isci.
2022.105469

Di Giovannantonio L., Acampora D., Omodei D., Nigro V., Barba P.,
Barbieri E., Chambers I., Simeone A. Direct repression of Nanog
and Oct4 by OTX2 modulates the contribution of epiblast-derived
cells to germline and somatic lineage. Development. 2021;148(10):
dev199166. doi 10.1242/dev.199166

Disteche C.M. Dosage compensation of the sex chromosomes and autosomes.
Semin Cell Dev Biol. 2016;56:9-18. doi 10.1016/j.semcdb.
2016.04.013

Duggal G., Warrier S., Ghimire S., Broekaert D., Van der Jeught M.,
Lierman S., Deroo T., … Heijmans B.T., Deforce D., De Sutter P.,
De Sousa Lopes S.C., Heindryckx B. Alternative routes to induce
naïve pluripotency in human embryonic stem cells. Stem Cells.
2015;33(9):2686-2698. doi 10.1002/stem.2071

Ee L.S., Medina-Cano D., Uyehara C.M., Schwarz C., Goetzler E., Salataj
E., Polyzos A., Madhuranath S., Evans T., Hadjantonakis A.K.,
Apostolou E., Vierbuchen T., Stadtfeld M. Transcriptional remodeling
by OTX2 directs specification and patterning of mammalian definitive
endoderm. bioRxiv. 2024. doi 10.1101/2024.05.30.596630

Fischer L.A., Meyer B., Reyes M., Zemke J.E., Harrison J.K., Park K.M.,
Wang T., Jüppner H., Dietmann S., Theunissen T.W. Tracking and
mitigating imprint erasure during induction of naïve human pluripotency
at single-cell resolution. Stem Cell Rep. 2025;20(3):102419.
doi 10.1016/j.stemcr.2025.102419

Gafni O., Weinberger L., Mansour A.A., Manor Y.S., Chomsky E., Ben-
Yosef D., Kalma Y., … Amann-Zalcenstein D., Benjamin S., Amit I.,
Tanay A., Massarwa R., Novershtern N., Hanna J.H. Derivation of
novel human ground state naïve pluripotent stem cells. Nature. 2013;
504(7479):282-286. doi 10.1038/nature12745

Grigor’eva E.V., Kopytova A.E., Yarkova E.S., Pavlova S.V., Sorogina
D.A., Malakhova A.A., Malankhanova T.B., Baydakova G.V.,
Zakharova E.Y., Medvedev S.P., Pchelina S.N., Zakian S.M.
Biochemical characteristics of iPSC-derived dopaminergic neurons
from N370S GBA variant carriers with and without Parkinson’s
disease. Int J Mol Sci. 2023;24(5):4437. doi 10.3390/ijms24054437

Grigor’eva E.V., Karapetyan L.V., Malakhova A.A., Medvedev S.P.,
Minina J.M., Hayrapetyan V.H., Vardanyan V.S., Zakian S.M.,
Arakelyan A., Zakharyan R. Generation of iPSCs from a patient with
the M694V mutation in the MEFV gene associated with Familial
Mediterranean fever and their differentiation into macrophages. Int J
Mol Sci. 2024;25(11):6102. doi 10.3390/ijms25116102

Gu M. Efficient differentiation of human pluripotent stem cells to
endothelial cells. Curr Protoc Hum Genet. 2018;98(1):e64. doi
10.1002/cphg.64

Guo G., von Meyenn F., Rostovskaya M., Clarke J., Dietmann S.,
Baker D., Sahakyan A., Myers S., Bertone P., Reik W., Plath K.,
Smith A. Epigenetic resetting of human pluripotency. Development.
2017;144(15):2748-2763. doi 10.1242/dev.146811

Guo G., Stirparo G.G., Strawbridge S.E., Spindlow D., Yang J.,
Clarke J., Dattani A., Yanagida A., Li M.A., Myers S., Özel B.N.,
Nichols J., Smith A. Human naïve epiblast cells possess unrestricted
lineage potential. Cell Stem Cell. 2021;28(6):1040-1056. doi
10.1016/j.stem.2021.02.025

Hossain I., Priam P., Reynoso S.C., Sahni S., Zhang X.X., Côté L., Doumat
J., Chik C., Fu T., Lessard J.A., Pastor W.A. ZIC2 and ZIC3 promote
SWI/SNF recruitment to safeguard progression towards human
primed pluripotency. Nat Commun. 2024;15(1):8539. doi 10.1038/
s41467-024-52431-1

Hu Z., Li H., Jiang H., Ren Y., Yu X., Qiu J., Stablewski A., Zhang B.,
Buck M., Feng J. Transient inhibition of mTOR in human pluripotent
stem cells enables robust formation of mouse-human chimeric
embryos. Sci Adv. 2020;6(20):eaaz0298. doi 10.1126/sciadv.aaz0298

Irie N., Surani M.A. Efficient induction and isolation of human primordial
germ cell-like cells from competent human pluripotent stem
cells. In: Buszczak M. (Ed.) Germline Stem Cells. Methods in Molecular
Biology. Vol. 1463. Humana Press, New York, 2016;217-226.
doi 10.1007/978-1-4939-4017-2_16

James D., Nam H.S., Seandel M., Nolan D., Janovitz T., Tomishima M.,
Studer L., Lee G., Lyden D., Benezra R., Zaninovic N., Rosenwaks
Z., Rabbany S.Y., Rafii S. Expansion and maintenance of human
embryonic stem cell-derived endothelial cells by TGFβ inhibition is Id1 dependent. Nat Biotechnol. 2010;28(2):161-166. doi
10.1038/nbt.1605

Khan S.A., Park K.M., Fischer L.A., Dong C., Lungjangwa T., Jimenez
M., Casalena D., Chew B., Dietmann S., Auld D.S., Jaenisch R.,
Theunissen T.W. Probing the signaling requirements for naïve human
pluripotency by high-throughput chemical screening. Cell Rep.
2021;35(11):109233. doi 10.1016/j.celrep.2021.109233

Kisa F., Shiozawa S., Oda K., Yoshimatsu S., Nakamura M., Koya I.,
Kawai K., Suzuki S., Okano H. Naïve-like ESRRB+ iPSCs with the
capacity for rapid neural differentiation. Stem Cell Rep. 2017;9(6):
1825-1838. doi 10.1016/j.stemcr.2017.10.008

Klepikova A., Nenasheva T., Sheveleva O., Protasova E., Antonov D.,
Gainullina A., Chikina E., Sakovnich O., Gerasimova T., Nikitina I.,
Shevalie D., Lyadova I. iPSC-derived macrophages: the differentiation
protocol affects cell immune characteristics and differentiation
trajectories. Int J Mol Sci. 2022;23(24):16087. doi 10.3390/
ijms232416087

Kopytova A.E., Rychkov G.N., Cheblokov A.A., Grigor’eva E.V.,
Nikolaev M.A., Yarkova E.S., Sorogina D.A., … Bezrukikh V.A.,
Salogub G.N., Zakharova E.Y., Pchelina S.N., Emelyanov A.K. Potential
binding sites of pharmacological chaperone NCGC00241607
on mutant β-glucocerebrosidase and its efficacy on patient-derived
cell cultures in Gaucher and Parkinson’s disease. Int J Mol Sci.
2023;24(10):9105. doi 10.3390/ijms24109105

Lee J.H., Laronde S., Collins T.J., Shapovalova Z., Tanasijevic B.,
McNicol
J.D., Fiebig-Comyn A., Benoit Y.D., Lee J.B., Mitchell
R.R.,
Bhatia M. Lineage-specific differentiation is influenced by state
of human pluripotency. Cell Rep. 2017;19(1):20-35. doi 10.1016/
j.celrep.2017.03.036

Liu X., Nefzger C.M., Rossello F.J., Chen J., Knaupp A.S., Firas J.,
Ford E., … Nilsson S.K., Schittenhelm R.B., Laslett A.L., Lister R.,
Polo J.M. Comprehensive characterization of distinct states of human
naïve pluripotency generated by reprogramming. Nat Methods.
2017;14(11):1055-1062. doi 10.1038/nmeth.4436

Malakhova A.A., Grigor’eva E.V., Pavlova S.V., Malankhanova T.B.,
Valetdinova K.R., Vyatkin Y.V., Khabarova E.A., Rzaev J.A., Zakian
S.M., Medvedev S.P. Generation of induced pluripotent stem
cell lines ICGi021-A and ICGi022-A from peripheral blood mononuclear
cells of two healthy individuals from Siberian population.
Stem Cell Res. 2020;48:101952. doi 10.1016/j.scr.2020.101952

Mathieu J., Detraux D., Kuppers D., Wang Y., Cavanaugh C., Sidhu S.,
Levy S., … Hawkins R.D., Moon R.T., Ware C.B., Paddison P.J.,
Ruohola-Baker H. Folliculin regulates mTORC1/2 and WNT pathways
in early human pluripotency. Nat Commun. 2019;10(1):632.
doi 10.1038/s41467-018-08020-0

Mazid M.A., Ward C., Luo Z., Liu C., Li Y., Lai Y., Wu L., … Maxwell
P.H., Xu X., Liu L., Li W., Esteban M.A. Rolling back human
pluripotent stem cells to an eight-cell embryo-like stage. Nature.
2022;605(7909):315-324. doi 10.1038/s41586-022-04625-0

Mekhoubad S., Bock C., De Boer A., Kiskinis E., Meissner A.,
Eggan
K. Erosion of dosage compensation impacts human iPSC
disease modeling. Cell Stem Cell. 2012;10(5):595-609. doi 10.1016/
j.stem.2012.02.014

Motosugi N., Sugiyama A., Okada C., Otomo A., Umezawa A.,
Akutsu H., Hadano S., Fukuda A. De-erosion of X chromosome
dosage compensation by the editing of XIST regulatory regions restores
the differentiation potential in hPSCs. Cell Rep Methods.
2022;2(12):100352. doi 10.1016/j.crmeth.2022.100352

Nadtochy J.A., Medvedev S.P., Grigor’eva E.V., Pavlova S.V.,
Minina J.M., Chechushkov A.V., Malakhova A.A., Kovalenko L.V.,
Zakian S.M. Transgenic iPSC lines with genetically encoded
MitoTimer to study mitochondrial biogenesis in dopaminergic
neurons with tauopathy. Biomedicines. 2025;13(3):550. doi 10.3390/
biomedicines13030550

Okae H., Toh H., Sato T., Hiura H., Takahashi S., Shirane K., Kabayama
Y., Suyama M., Sasaki H., Arima T. Derivation of human trophoblast
stem cells. Cell Stem Cell. 2018;22(1):50-63. doi 10.1016/
j.stem.2017.11.004

Ozaki H., Suga H., Sakakibara M., Soen M., Miyake N., Miwata T.,
Taga S., … Iguchi G., Takahashi Y., Muguruma K., Inoue H.,
Arima H. Differentiation of human induced pluripotent stem cells
into hypothalamic vasopressin neurons with minimal exogenous
signals and partial conversion to the naïve state. Sci Rep. 2022;12(1):
17381. doi 10.1038/s41598-022-22405-8

Patel S., Bonora G., Sahakyan A., Kim R., Chronis C., Langerman J.,
Fitz-Gibbon S., … Ardehali R., Pellegrini M., Lowry W.E., Clark A.T.,
Plath K. Human embryonic stem cells do not change their X inactivation
status during differentiation. Cell Rep. 2017;18(1):54-67. doi
10.1016/j.celrep.2016.11.054

Pavlova S.V., Shulgina A.E., Minina J.M., Zakian S.M., Dementyeva
E.V. Generation of isogenic iPSC lines for studying the effect of
the p.N515del (c.1543_1545delAAC) variant on MYBPC3 function
and hypertrophic cardiomyopathy pathogenesis. Int J Mol Sci.
2024a;25(23):12900. doi 10.3390/ijms252312900

Pavlova S.V., Shulgina A.E., Zakian S.M., Dementyeva E.V. Studying
pathogenetic contribution of a variant of unknown significance,
p.M659I (c.1977G > A) in MYH7, to the development of hypertrophic
cardiomyopathy using CRISPR/Cas9-engineered isogenic induced
pluripotent stem cells. Int J Mol Sci. 2024b;25(16):8695. doi
10.3390/ijms25168695

Pham T.X.A., Panda A., Kagawa H., To S.K., Ertekin C., Georgolopoulos
G., van Knippenberg S.S.F.A., … Lluis F., David L.,
Rivron N., Balaton B.P., Pasque V. Modeling human extraembryonic
mesoderm cells using naïve pluripotent stem cells. Cell Stem Cell.
2022;29(9):1346-1365.e10. doi 10.1016/j.stem.2022.08.001

Raposo A.C., Caldas P., Jeremias J., Arez M., Cazaux Mateus F., Barbosa
P., Sousa-Luís R., … Mupo A., Eckersley-Maslin M., Casanova
M., Grosso A.R., da Rocha S.T. Gene reactivation upon erosion
of X chromosome inactivation in female hiPSCs is predictable
yet variable and persists through differentiation. Stem Cell Rep.
2025;20(5):102472. doi 10.1016/j.stemcr.2025.102472

Rezvova M.A., Ovcharenko E.A., Klyshnikov K.Y., Glushkova T.V.,
Kostyunin A.E., Shishkova D.K., Matveeva V.G., Velikanova E.A.,
Shabaev A.R., Kudryavtseva Y.A. Electrospun bioresorbable polymer
membranes for coronary artery stents. Front Bioeng Biotechnol.
2024;12:1440181. doi 10.3389/fbioe.2024.1440181

Rostovskaya M. Capacitation of human naïve pluripotent stem cells.
In: Rugg-Gunn P. (Ed.) Human Naïve Pluripotent Stem Cells.
Methods
in Molecular Biology. Vol. 2416. Humana, New York,
2022;117-131. doi 10.1007/978-1-0716-1908-7_9

Rostovskaya M., Stirparo G., Smith A. Capacitation of human naïve
pluripotent stem cells for multi-lineage differentiation. Development.
2019;146(7):dev172916. doi 10.1242/dev.172916

Sahakyan A., Kim R., Chronis C., Sabri S., Bonora G., Theunissen T.,
Kuoy E., Langerman J., Clark A., Jaenisch R., Plath K. Human
naïve
pluripotent stem cells model X chromosome dampening and
X inactivation. Cell Stem Cell. 2017;20(1):87-101. doi 10.1016/
j.stem.2016.10.006

Shevchenko A.I., Arssan A.M., Zakian S.M., Zakharova I.S. Chemokine
CCL2 activates hypoxia response factors regulating pluripotency
and directed endothelial differentiation of human pluripotent
stem cells. Russ J Dev Biol. 2023;54(2):134-146. doi 10.1134/
s1062360423020054

Shevchenko A.I., Arssan A.M., Zakharova I.S. Towards the generation
of safe naïve human pluripotent cell lines. Tomsk State Univ J Biol.
2025;69:184-193. doi 10.17223/19988591/69/21 (in Russian)

Sheveleva O., Protasova E., Nenasheva T., Butorina N., Melnikova V.,
Gerasimova T., Sakovnich O., Kurinov A., Grigor’eva E., Medvedev
S., Lyadova I. A model of iPSC-derived macrophages with
TNFAIP3 overexpression reveals the peculiarities of TNFAIP3 protein
expression and function in human macrophages. Int J Mol Sci
2023;24(16):12868. doi 10.3390/ijms241612868

Sheveleva O., Protasova E., Grigor’eva E., Butorina N., Kuziaeva V.,
Antonov D., Melnikova V., Medvedev S., Lyadova I. The generation
of genetically engineered human induced pluripotent stem
cells overexpressing IFN-β for future experimental and clinically oriented studies. Int J Mol Sci. 2024;25(22):12456. doi 10.3390/
ijms252212456

Stadtfeld M., Hochedlinger K. Induced pluripotency: history, mechanisms,
and applications. Genes Dev. 2010;24(20):2239-2263. doi
10.1101/gad.1963910

Szczerbinska I., Gonzales K.A.U., Cukuroglu E., Ramli M.N.B.,
Lee B.P.G., Tan C.P., Wong C.K., Rancati G.I., Liang H., Göke J.,
Ng H.H., Chan Y.S. A chemically defined feeder-free system for
the establishment and maintenance of the human naïve pluripotent
state. Stem Cell Rep. 2019;13(4):612-626. doi 10.1016/j.stemcr.
2019.08.005

Takashima Y., Guo G., Loos R., Nichols J., Ficz G., Krueger F., Oxley
D., Santos F., Clarke J., Mansfield W., Reik W., Bertone P.,
Smith A. Resetting transcription factor control circuitry toward
ground-state pluripotency in human. Cell. 2014;158(6):1254-1269.
doi 10.1016/j.cell.2014.08.029

Theunissen T.W., Powell B.E., Wang H., Mitalipova M., Faddah D.A.,
Reddy J., Fan Z.P., … Gao Q., Dawlaty M.M., Young R.A.,
Gray N.S., Jaenisch R. Systematic identification of culture conditions
for induction
and maintenance of naïve human pluripotency.
Cell Stem Cell. 2014;15(4):471-487. doi 10.1016/j.stem.2014.07.002

Theunissen T.W., Friedli M., He Y., Planet E., O’Neil R.C., Markoulaki
S., Pontis J., … Drotar J., Lungjangwa T., Trono D., Ecker J.R.,
Jaenisch R. Molecular criteria for defining the naïve human pluripotent
state. Cell Stem Cell. 2016;19(4):502-515. doi 10.1016/
j.stem.2016.06.011

Ustyantseva E., Pavlova S.V., Malakhova A.A., Ustyantsev K., Zakian
S.M., Medvedev S.P. Oxidative stress monitoring in iPSC-derived
motor neurons using genetically encoded biosensors of H2O2.
Sci Rep. 2022;12(1):8928. doi 10.1038/s41598-022-12807-z

Valamehr B., Robinson M., Abujarour R., Rezner B., Vranceanu F.,
Le T., Medcalf A., Lee T., Fitch M., Robbins D., Flynn P. Platform
for induction and maintenance of transgene-free hiPSCs resembling
ground state pluripotent stem cells. Stem Cell Rep. 2014;2(3):366-
381. doi 10.1016/j.stemcr.2014.01.014

Vallot C., Ouimette J.F., Makhlouf M., Féraud O., Pontis J., Côme J.,
Martinat C., Bennaceur-Griscelli A., Lalande M., Rougeulle C.
Erosion of X chromosome inactivation in human pluripotent cells
initiates with XACT coating and depends on a specific heterochromatin
landscape. Cell Stem Cell. 2015;16(5):533-546. doi 10.1016/
j.stem.2015.03.016

Vallot C., Patrat C., Collier A.J., Huret C., Casanova M., Liyakat
Ali T.M., Tosolini M., Frydman N., Heard E., Rugg-Gunn P.J.,
Rougeulle C. XACT noncoding RNA competes with XIST in the control
of X chromosome activity during human early development. Cell
Stem Cell. 2017;20(1):102-111. doi 10.1016/j.stem.2016.10.014

Vaskova E.A., Medvedev S.P., Sorokina A.E., Nemudryy A.A., Elisaphenko
E.A., Zakharova I.S., Shevchenko A.I., Kizilova E.A., Zhelezova
A.I., Evshin I.S., Sharipov R.N. Transcriptome characteristics
and X-chromosome inactivation status in cultured rat pluripotent
stem cells. Stem Cells Dev. 2015;24(24):2912-2924. doi 10.1089/
scd.2015.0204

Ware C.B., Nelson A.M., Mecham B., Hesson J., Zhou W., Jonlin E.C.,
Jimenez-Caliani A.J., … Blau C.A., Treuting P.M., Hawkins R.D.,
Cirulli V., Ruohola-Baker H. Derivation of naïve human embryonic
stem cells. Proc Natl Acad Sci USA. 2014;111(12):4484-4489. doi
10.1073/pnas.1319738111

Warr N., Powles-Glover N., Chappell A., Robson J., Norris D.,
Arkell R. Zic2-associated holoprosencephaly is caused by a transient
defect in the organizer region during gastrulation. Hum Mol Gen.
2008;17(19):2986-2996. doi 10.1093/hmg/ddn197

Welling M., Chen H.H., Muñoz J., Musheev M.U., Kester L., Junker J.P.,
Mischerikow N., Arbab M., Kuijk E., Silberstein L., Kharchenko P.V.
DAZL regulates Tet1 translation in murine embryonic stem cells.
EMBO Rep. 2015;16(7):791-802. doi 10.15252/embr.201540538

Yarkova E.S., Grigor’eva E.V., Medvedev S.P., Tarasevich D.A.,
Pavlova S.V., Valetdinova K.R., Minina J.M., Zakian S.M., Malakhova
A.A. Detection of ER stress in iPSC-derived neurons carrying
the p. N370S mutation in the GBA1 gene. Biomedicines. 2024;
12(4):744. doi 10.3390/biomedicines12040744

Yu L., Wei Y., Sun H.X., Mahdi A.K., Pinzon Arteaga C.A., Sakurai M.,
Schmitz D.A., … Okamura D., Mutto A.A., Gu Y., Ross P.J., Wu J.
Derivation of intermediate pluripotent stem cells amenable to primordial
germ cell specification. Cell Stem Cell. 2021;28(3):550-567.
doi 10.1016/j.stem.2020.11.003

Zakharova I.S., Zhiven’ M.K., Saaya S.B., Shevchenko A.I., Smirnova
A.M., Strunov A., Karpenko A.A., Pokushalov E.A., Ivanova
L.N., Makarevich P.I., Parfyonova Y.V. Endothelial and smooth
muscle cells derived from human cardiac explants demonstrate angiogenic
potential and suitable for design of cell-containing vascular
grafts. J Transl Med. 2017;15(1):54. doi 10.1186/s12967-017-1156-1Zakharova I., Saaya S., Shevchenko A., Stupnikova A., Zhiven’ M.,
Laktionov P., Stepanova A., … Zavjalov E., Chernyavsky A., Romanov
A., Karpenko A., Zakian S. Mitomycin-treated endothelial
and smooth muscle cells suitable for safe tissue engineering approaches.
Front Bioengin Biotechnol. 2022;10:772981. doi 10.3389/
fbioe.2022.772981

Zakharova I.S., Shevchenko A.I., Arssan M.A., Sleptcov A.A., Nazarenko
M.S., Zarubin A.A., Zheltysheva N.V., … Saaya S.B.,
Ezhov M.V., Kukharchuk V.V., Parfyonova Y.V., Zakian S.M. iPSCderived
endothelial cells reveal LDLR dysfunction and dysregulated
gene expression profiles in familial hypercholesterolemia. Int J Mol
Sci. 2024;25(2):689. doi 10.3390/ijms25020689

Zimmerlin L., Park T.S., Huo J.S., Verma K., Pather S.R., Talbot C.C.,
Jr., Agarwal J., … Cope L., Canto-Soler M.V., Friedman A.D., Baylin
S.B., Zambidis E.T. Tankyrase inhibition promotes a stable human
naïve pluripotent state with improved functionality. Development.
2016;143(23):4368-4380. doi 10.1242/dev.138982

